# Translating Recommendations Into Reality: Community Voices

**Published:** 2007-06-15

**Authors:** Katie Adamson, Clark Baker, E. Yvonne Lewis

**Affiliations:** YMCA of the USA; YMCA of the Greater Houston Area, Houston, Tex; Faith Access to Community Economic Development (a REACH partner), Flint, Mich

Thousands of communities throughout the country are served by Young Men's Christian Associations (YMCAs) and Racial and Ethnic Approaches to Community Health (REACH) 2010 to build healthier lives. We salute the Centers for Disease Control and Prevention (CDC) for recognizing the importance of integrating voices from all communities, including those with mixed incomes, differing races and ethnicities, and diverse cultures.

YMCAs seek to build a healthy spirit, mind, and body for all by uniting men, women, and children of all ages, faiths, backgrounds, abilities, and income levels from urban centers to small towns. REACH programs, funded by CDC, mobilize community coalitions in designing, implementing, and evaluating community-driven, evidence-based strategies to fit their unique needs, demonstrating that these programs can reduce health disparities.

YMCAs and REACH believe that only an approach involving both science and real-world practice can curb chronic disease and effect cultural change. With that in mind, we want to address a few of the recommendations of the National Expert Panel on Community Health Promotion (1):

- *Enhanced surveillance.* YMCA's Activate America project and REACH 2010 have identified the need to measure not only risk factors but also the opportunities and needs of communities to improve their populations' health. Community capacity can be measured by the availability of, access to, and trust in facilities such as community centers, parks, schools, grocery stores, farmers' markets, and safe streets, and the presence of committed and engaged leaders in those communities. Thus, YMCAs and REACH support the recent Institute of Medicine recommendation (2) to develop a community healthy living index tool with modules for 1) the availability, accessibility, attractiveness, affordability, and safety of places for physical activity and healthier food choices; 2) the involvement of community organizations; and 3) the efficacy of a community's efforts to encourage healthy lifestyles. YMCA of the USA has begun to develop such a tool and looks forward to working with REACH and other community-based organizations and CDC in further developing and implementing this tool.
- *Promoting community-based participatory research.* We believe this approach can produce true knowledge of local assets, strengths, and weaknesses. REACH is grounded in this philosophy. Promoting true partnership among academic institutions and community-based organizations should produce better results in programs and policy strategies and deeper ownership of this work in communities. However, these results cannot be achieved overnight. Years are required to build effective relationships with community residents and to earn their trust. This is an ongoing challenge. Community voices must be heard and respected.
- *Promote training and capacity building for community health approaches.* This is essential to helping communities replicate and expand best practices. YMCAs have a long history of training staff and volunteers to deliver programs that sustain and extend the reach of their work. During the last 3 years YMCAs trained community volunteers to deliver effective healthy-eating and active-living curricula to children in after-school and camping programs. The Houston YMCA recruited and trained bilingual staff and volunteers to deliver programs through Apartment Outreach, which operates in 16 residential sites and provides health education and risk assessment programs, recreational activities, and other programs. In REACH programs, community health advisors have led programs that reduced disparities in incidence of high blood glucose levels, incidence of high blood pressure, infant mortality rates, and breast and cervical cancer screening rates. For example, since the Genesee County (Michigan) REACH initiative began in 1999, African American infant mortality rates have decreased from 3.18 per 1000 live births to 2.41 per 1000.
- *Maximize the impact of federal resources through greater collaboration and coordination among agencies* (and, we would add, community-based organizations). Much of the work to address chronic disease at all levels is performed in silos. Fragmentation is driven by dollars, information overload, and time constraints that make connecting difficult. The YMCA Activate America: Pioneering Healthier Communities project has quickened the pace of policy and environmental change by funding teams of local leaders to direct change efforts. A recent study by the General Accounting Office (3) identified REACH as one of the federal government's most successful and viable efforts to actively engage affected communities. Multisector collaboration at the federal, state, and community levels is essential if these programs are to be successful.

The federal government and private foundations have made substantial investments in communities. Funded communities can become anchor sites for training others, sharing promising practices, and building capacity. Primary to the success of these efforts is the need to respect the differences and unique strengths of communities (both urban and rural), engage community leaders to drive change at the local level, and continue to work with CDC to build an infrastructure that supports and sustains healthy communities.
